# Clinical Insights Into Bilateral Mandibular Angle Fractures: Our Experience

**DOI:** 10.7759/cureus.104979

**Published:** 2026-03-10

**Authors:** Konda Sireesha Reddy, Suhas Mall, Vempalli Nagasubramanyam, Vivek Kumar, Takshak Shankar

**Affiliations:** 1 Department of Trauma and Emergency Medicine, All India Institute of Medical Sciences, Gorakhpur, Gorakhpur, IND; 2 Department of Emergency Medicine, All India Institute of Medical Sciences, Rishikesh, Rishikesh, IND

**Keywords:** bilateral mandibular angle fracture, mandibular angle, mandibular angle fracture management, orif mandible, road traffic injuries

## Abstract

Bilateral mandibular angle fractures are uncommon but present a significant challenge in terms of management for maxillofacial surgeons. We present a case of an adolescent male who presented to the emergency department after a road traffic accident with isolated bilateral mandibular angle fractures, with a significant displacement on the left side. The patient was treated using rigid fixation for the more displaced left angle and non-rigid fixation for the right side, which led to a stable reduction without complications.

## Introduction

Bilateral mandibular angle fractures are considered a rare traumatic occurrence [1]. Even though extensive research has been done on unilateral angle of mandible fractures, the data on bilateral mandibular angle fractures are still limited [2-4].

Anatomically, the mandibular angle, subcondylar region, and mental area are the most vulnerable, making mandibular angle fractures relatively common [5,6]. The susceptibility of this area is often attributed to three main factors: the presence of the third molar, the curvature at the mandibular angle, and the thin cross-sectional area at the angle [6,7].

Mandibular angle fractures can occur alone or in combination with other fractures, such as those of the symphysis or condyles, whereas Bilateral mandibular angle fractures are less frequent [1]. Cillo Jr. and Ellis III reported 33 cases out of 1,565 patients (2.1%) over a 20-year period, while Boffano and Roccia recorded 8 cases out of 635 patients over 8 years [8,9]. Despite this, there is limited literature on the optimal treatment for mandibular angle fractures, particularly concerning bilateral fractures. In this report, we describe the case of an early adolescent male who sustained isolated bilateral mandibular angle fractures following a road traffic accident.

## Case presentation

An early adolescent male was brought to the Department of Trauma and Emergency Medicine after being involved in a road traffic accident, where a four-wheeler collided with the two-wheeler he was riding as a pillion passenger. The patient was evaluated according to the Advanced Trauma Life Support (ATLS) protocol and was conscious, with a Glasgow Coma Scale (GCS) score of 15/15. His primary complaints included pain, swelling in the lower jaw, and difficulty fully opening or closing his mouth. Additionally, he had an injury to his left knee, which was managed by the orthopedic team.

Upon clinical examination, the patient was stable, with no signs of neurological, chest, or abdominal injury. The mandible was palpated for fractures and midline deviation, revealing fractures on both the left and right mandibular angles, along with an anterior open bite. The left mandible had a 3 cm laceration on its inferior border, with tenderness noted at both mandibular angles and malocclusion observed, suggesting an open fracture. Intraorally, a gingival laceration was noted between the second and third lower molars on the left side. The left mandibular third molar was loose, but the tooth root appeared intact. The right side had a closed mandibular angle fracture with no injury to the third molar.

**Figure 1:**
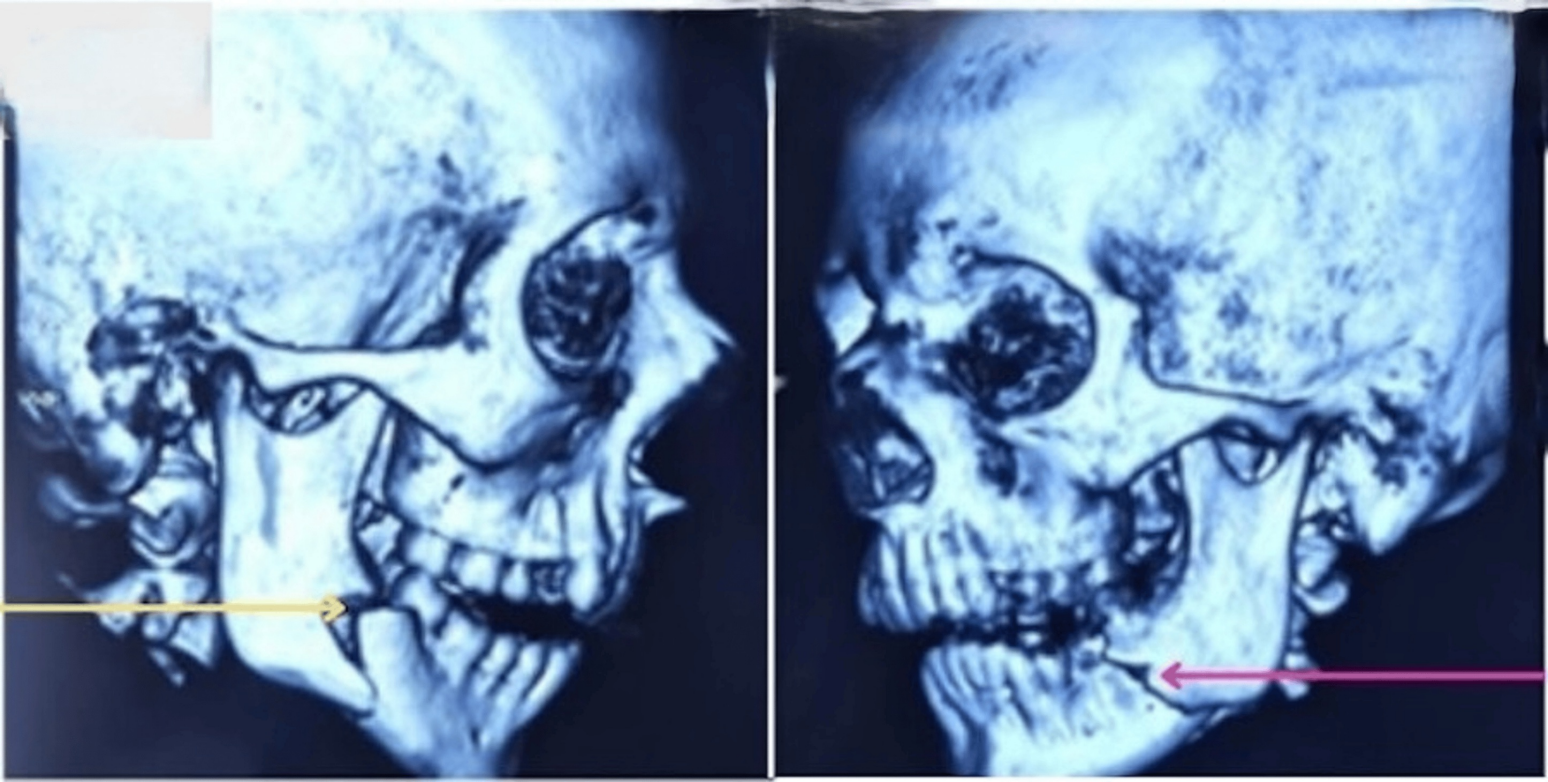
Preoperative non-contrast CT of the head with 3D facial reconstruction showing a right mandibular angle fracture (yellow arrow) and left mandibular angle fracture (red arrow).

After stabilization, the patient underwent a CT scan with 3D reconstruction, which confirmed bilateral mandibular angle fractures, with significant displacement on the left side (Figure 1).

As initial emergency management, the patient received intravenous fluids, tetanus toxoid vaccination, analgesics, antacids, and broad-spectrum antibiotics, including IV amoxicillin clavulanate and 500 mg of IV metronidazole. The wound was irrigated with normal saline, and the left lower jaw laceration was sutured with nylon 4.0 sutures, ensuring hemostasis. All investigations relevant to fitness for anesthesia, including complete blood count, liver function tests, kidney function tests, viral markers, electrocardiogram, and chest X-ray, were performed, and the results were within normal limits.

After ensuring that the patient was adequately resuscitated and clinically stable, he was planned for open reduction and internal fixation of fractures (ORIF) under general anesthesia. Bimaxillary arch bars were placed, and temporary intermaxillary fixation (IMF) was performed to check and correct the occlusion. The left mandibular angle, which was more displaced, was approached through a combined extraoral and intraoral incision. The fracture was reduced, and fixation was achieved using two four-hole titanium miniplates, one on the superior and one on the inferior border. For the less displaced right mandibular angle, an intraoral incision was used to place a single four-hole titanium miniplate along the external oblique ridge. After fracture fixation, the correct occlusal alignment was confirmed, and intraoral incisions on both sides were closed with absorbable sutures. The extraoral laceration was closed in layers using a combination of absorbable and non-absorbable sutures.

**Figure 2:**
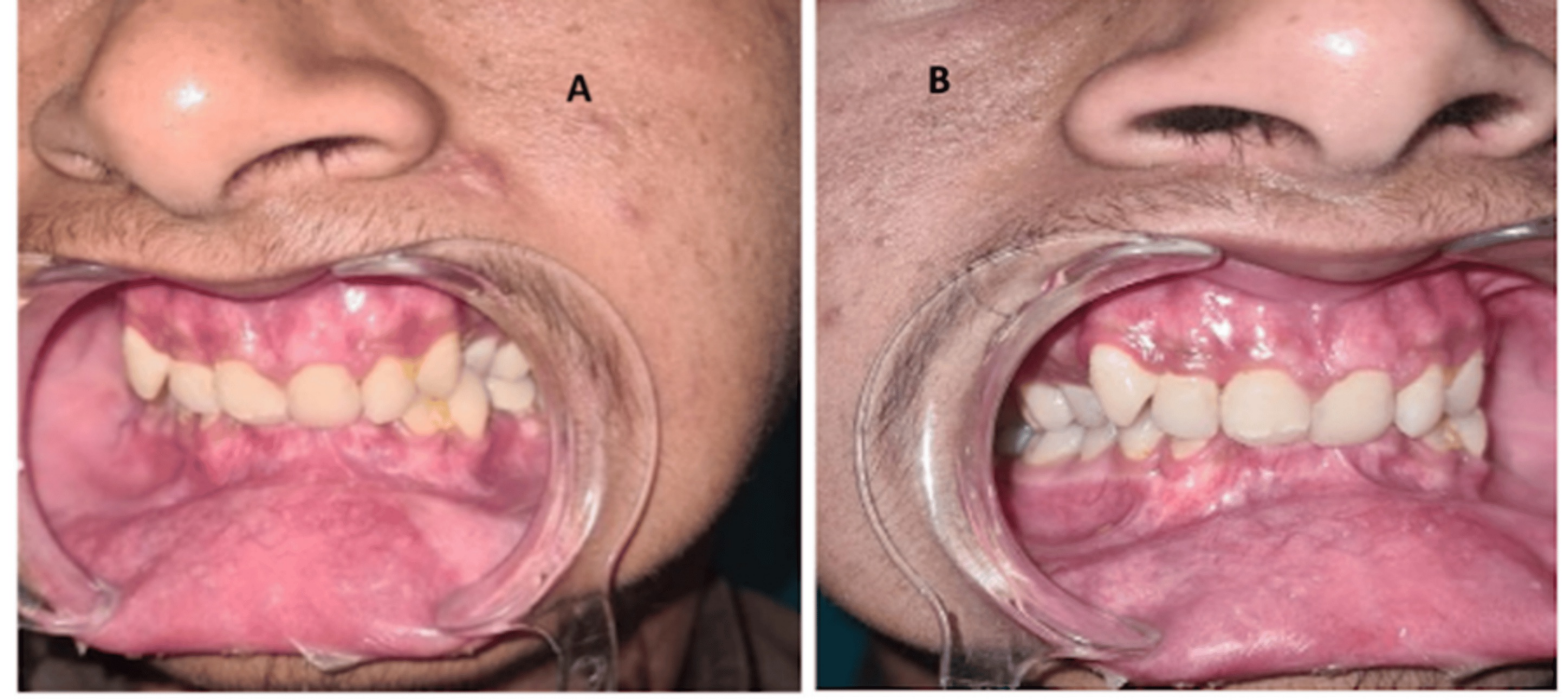
Postoperative occlusion on the left side (A) and the right side (B).

The patient received IV antibiotics postoperatively for three days, and IMF rubber bands were placed on the next postoperative day (POD-1) to achieve stable occlusion. The patient was discharged after three days with IMF in place, to be followed up after 14 days. He was prescribed a non-chewing diet, oral antibiotics, analgesics, and mouthwash for oral hygiene maintenance. The postoperative recovery was uneventful, and stable occlusion was noted on the 14-day follow-up, after which the IMF and arch bars were removed (Figure 2).

**Figure 3:**
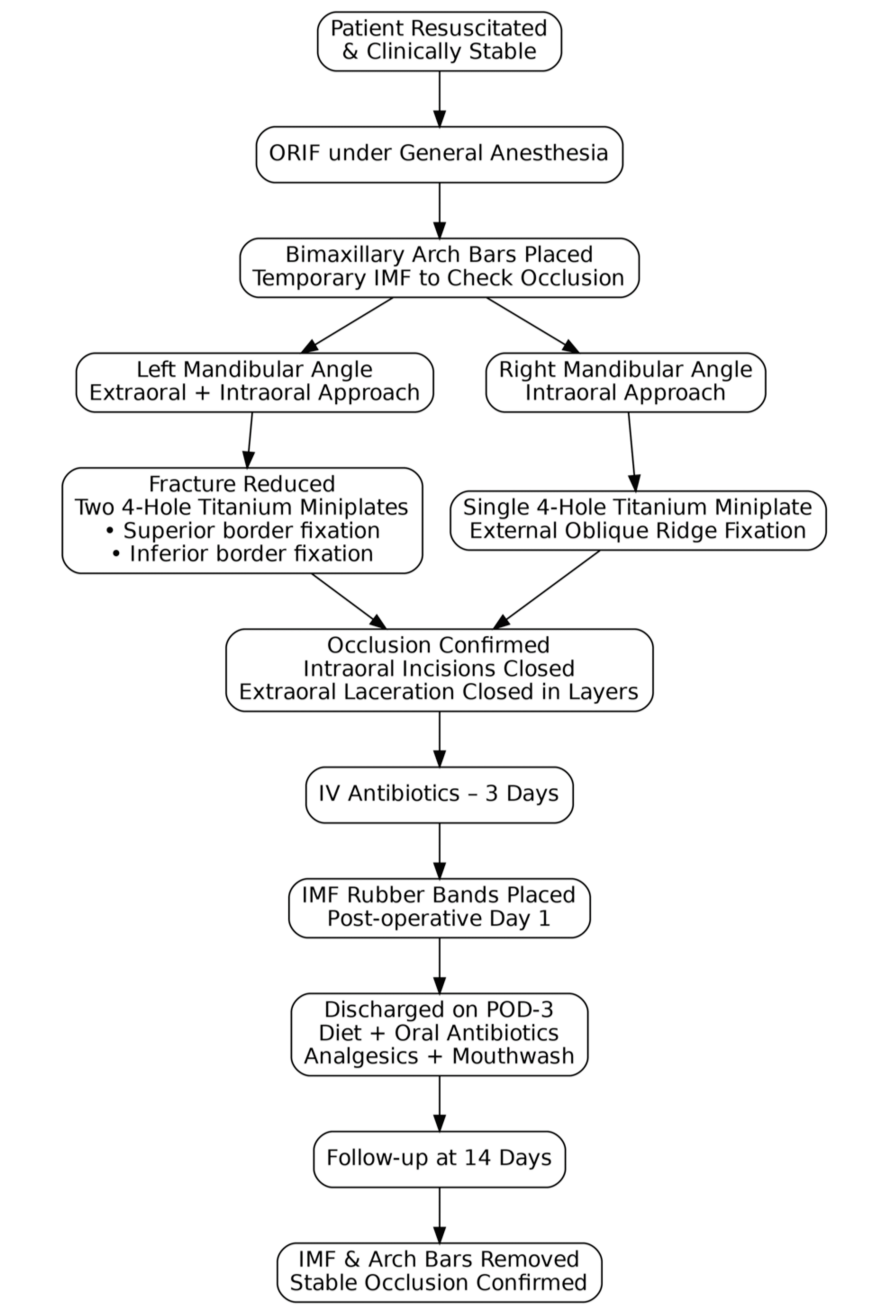
Patient management flowchart. ORIF: open reduction and internal fixation; IMF: intermaxillary fixation; POD: postoperative day

A flowchart of the patient management is presented in Figure 3.

**Figure 4:**
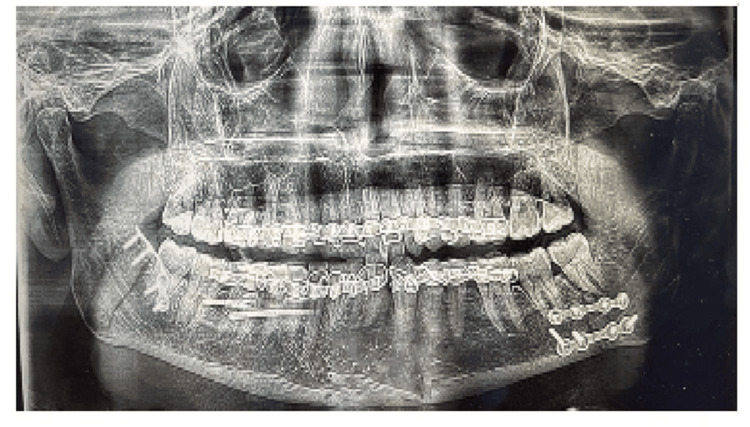
Postoperative panoramic teleradiograph at the six-month follow-up.

The patient was followed up for six months, and no complications were noted. A postoperative panoramic teleradiograph was taken during the follow-up period (Figure 4).

## Discussion

Mandibular fractures are the most common type of maxillofacial fractures. According to a study by Olson et al., 24.5% of all mandibular fractures occur at the angle of the mandible, with the mandibular angle being the second most frequent site for fractures, following the condyle [3,10].

Bilateral fractures of the mandibular angles are relatively rare and have been less thoroughly studied. Bilateral mandibular angle fractures commonly affect males in their third decade of life and are often associated with impacted mandibular third molars, which reduce the resistance to fracture [11]. The most common mechanisms of injury include road traffic accidents, interpersonal violence, sports injuries, and falls [1,7,12]. CT remains the gold standard for diagnosing mandibular fractures, including Bilateral mandibular angle fractures [13].

Mandibular angle fractures are complex due to the high likelihood of complications and the challenging surgical approach [14]. Various fixation techniques for unilateral mandibular angle fractures, ranging from non-rigid to rigid fixation, have been proposed, including lag screws, large reconstruction bone plates, compression plates along the lower border, and miniplates at the inferior or superior borders. Studies have shown that a single superior border plate offers stability for unilateral angle fractures, allowing for proper healing of the fracture [1,15]. The surgical approach for mandibular fractures varies. Some studies advocate for a transbuccal approach due to its lower complication rate, while others prefer an intraoral approach for the Champy technique, which promotes quicker recovery and reduces facial nerve injury as well as avoids facial scars [1,16].

Bilateral mandibular angle fractures require accurate and prompt intervention to prevent possible complications such as infection, malocclusion, and hardware failure [1, 17]. Cillo Jr. and Ellis III highlighted that fixation requirements for bilateral fractures are not adequately addressed in standard texts [1]. A recent study by Young et al. suggested that non-rigid fixation for both sides of bilateral fractures can achieve outcomes similar to those with rigid fixation on one side [16]. A recent literature review by Champy et al. supported the use of the Champy technique or two miniplates for mandibular angle fractures [18]. According to Cillo Jr. and Ellis III, bilateral fractures are less stable than unilateral fractures, with the stability after fixation being influenced by the degree of displacement [1].

Following resuscitation and stabilization of the trauma patient, definitive fracture management by either closed or open reduction is performed [3,14]. In our patient, initial assessment and management were performed according to ATLS protocols with definitive treatment based on current literature [1,6,12]. The diagnosis was confirmed with a 3D CT scan, and the more displaced left angle was approached using both an intraoral incision and an extraoral laceration wound. The non-displaced right angle was approached via an intraoral access. The left side fracture received rigid fixation with two miniplates, while the right side was treated with non-rigid fixation using a single plate along the superior border.

An important consideration in these cases is the management of the third molar (M3) within the fracture line. Literature generally favors preserving the third molar unless there are specific indications for extraction, such as impacted teeth with cysts, pericoronitis, fractured roots, or exposed roots, and those obstructing the correct reduction of the fracture [19,20]. In our case, the third molars were retained as they did not interfere with fracture reduction, and there were no signs of cystic lesions or infection. Prophylactic antibiotics were given, and the patient underwent open reduction.

## Conclusions

Bilateral mandibular angle fractures present a challenging clinical scenario, with muscular forces acting in opposing directions on three separate fracture fragments. Stabilization methods that adhere to biomechanical principles are essential to prevent complications such as non-union and malunion. The main objectives of mandibular fracture treatment are to restore occlusion and ensure proper fracture healing. Our approach, using rigid fixation for the more displaced left angle and non-rigid fixation for the right side, led to a stable reduction without complications in this patient. However, additional studies are required before any reliable recommendations can be made.
